# Transcriptomic Profiling of Gene Expression Associated with Granulosa Cell Tumor Development in a Mouse Model

**DOI:** 10.3390/cancers14092184

**Published:** 2022-04-27

**Authors:** Nan Ni, Xin Fang, Destiny A. Mullens, James J. Cai, Ivan Ivanov, Laurent Bartholin, Qinglei Li

**Affiliations:** 1 Department of Veterinary Integrative Biosciences, Texas A&M University, College Station, TX 77843, USA; nni@cvm.tamu.edu (N.N.); xfang@cvm.tamu.edu (X.F.); jcai@cvm.tamu.edu (J.J.C.); 2 Department of Veterinary Physiology and Pharmacology, Texas A&M University, College Station, TX 77843, USA; destiny_mullens@tamu.edu (D.A.M.); iivanov@cvm.tamu.edu (I.I.); 3 INSERM U1052, CNRS UMR5286, Centre de Recherche en Cancérologie de Lyon, Université Lyon 1, F-69000 Lyon, France; laurent.bartholin@lyon.unicancer.fr; 4 Centre Léon Bérard, F-69008 Lyon, France

**Keywords:** granulosa cell tumors, TGFBR1, gene ontology, signaling pathways

## Abstract

**Simple Summary:**

Ovarian granulosa cell tumors are rare ovarian tumors, with poorly understood etiology. The aim of this study is to define the molecular changes in ovarian granulosa cell tumors. We used a granulosa cell tumor mouse model that contains dysregulated transforming growth factor β signaling. Using RNA-sequencing technology, we identified differentially expressed genes between tumor tissues and normal controls. In addition, comparative analyses have been performed to reveal common genes altered in ovarian granulosa cell tumors between the mouse and the human. This study has revealed the molecular signature of ovarian granulosa cell tumors in a mouse model. The results are potentially important to understand cell signaling events that regulate the development of a class of rare ovarian tumors.

**Abstract:**

Ovarian granulosa cell tumors (GCTs) are rare sex cord-stromal tumors, accounting for ~5% ovarian tumors. The etiology of GCTs remains poorly defined. Genetically engineered mouse models are potentially valuable for understanding the pathogenesis of GCTs. Mice harboring constitutively active TGFβ signaling (TGFBR1-CA) develop ovarian GCTs that phenocopy several hormonal and molecular characteristics of human GCTs. To determine molecular alterations in the ovary upon TGFβ signaling activation, we performed transcriptomic profiling of gene expression associated with GCT development using ovaries from 1-month-old TGFBR1-CA mice and age-matched controls. RNA-sequencing and bioinformatics analysis coupled with the validation of select target genes revealed dysregulations of multiple cellular events and signaling molecules/pathways. The differentially expressed genes are enriched not only for known GCT-related pathways and tumorigenic events but also for signaling events potentially mediated by neuroactive ligand-receptor interaction, relaxin signaling, insulin signaling, and complements in TGFBR1-CA ovaries. Additionally, a comparative analysis of our data in mice with genes dysregulated in human GCTs or granulosa cells overexpressing a mutant FOXL2, the genetic hallmark of adult GCTs, identified some common genes altered in both conditions. In summary, this study has revealed the molecular signature of ovarian GCTs in a mouse model that harbors the constitutive activation of TGFBR1. The findings may be further exploited to understand the pathogenesis of a class of poorly defined ovarian tumors.

## 1. Introduction

Ovarian granulosa cell tumors (GCTs) are the most common sex cord-stromal tumors, accounting for ~5% of ovarian tumors [1]. Although GCTs are of low malignant potential, these tumors are associated with a long natural history, frequent recurrence, and death [2]. The poorly understood etiology of GCTs creates a significant technical barrier for developing effective therapies. Genetically engineered mouse models targeting transforming growth factor β (TGFβ) superfamily signaling and Wnt/β-catenin have been developed to understand the molecular events in granulosa cell tumorigenesis [3,4,5]. Elegant work from the Richards’ group has pointed to the cooperative functions of forkhead box protein O1 (*Foxo1*), *Foxo3*, and phosphatase and tensin homolog (*Pten*) in GCT development [6].

TGFβ superfamily proteins are key regulators of cell proliferation, differentiation, and invasion [7]. The canonical TGFβ signaling requires the type 2 and type 1 receptors (TGFBR2/TGFBR1) and SMAD transducers (SMAD2/3 and 4). Accumulating evidence indicates a critical role of TGFβ superfamily signaling in ovarian GCT development [7]. Treatment of human COV434 cells with TGFβ1 enhances cell viability and reduces apoptosis, favoring tumor development [8]. *Smad*1/5 ovarian-specific knockout mice develop GCTs, with the activation of TGFβ signaling [4]. Of note, the development of GCTs is attenuated upon *Smad4* deletion in *Smad*1/5 conditionally ablated mice, arguing for a role of SMAD4-mediated TGFβ signaling in ovarian GCTs [8]. Direct evidence supporting a role of TGFβ signaling activation in ovarian GCT formation stems from our recently developed mouse model that harbors a constitutively active TGFBR1 in the ovary [9]. Ovarian tumors in these mice exhibit pathological, hormonal, and molecular similarities to human GCTs [9]. 

A cornerstone study has revealed the pathognomonic mutation of forkhead box L2 (FOXL2^C134W^) in adult ovarian GCTs [10]. It was recently demonstrated that FOXL2^C134W^/SMAD3 overexpression impacts the expression of genes including FOXL2 targets and neoplastic pathways [11]. Recent progresses have also been made on the discovery of GCT biomarkers and the development of new therapeutic strategies. One study demonstrated the detection of *FOXL2* mutant circulating tumor DNA in the plasma of ~79% adult GCT patients, making it an attractive biomarker [12]. In another study, targeting FOXL2 using a Foxl2-tetanus toxin vaccine leads to a therapeutic benefit [13]. TGFβ signaling appears to interact with FOXL2 during GCT development [14]. The mutant FOXL2^C134W^ forms a complex with SMAD4-SMAD2/3 and binds a unique hybrid DNA motif, resulting in the change of chromatin state and the transcription of genes associated with tumor development [14]. This evidence underscores the importance of TGFβ signaling in the pathogenesis of ovarian GCTs and suggests a therapeutic potential for targeting TGFβ signaling to treat ovarian GCTs.

To further understand how TGFβ signaling activation promotes ovarian GCT development, we performed transcriptomic analysis by RNA sequencing (RNA-seq) using ovaries from mice harboring constitutively active TGFBR1 and age-matched controls during early tumor development. Bioinformatics analysis of the transcriptomic profiles coupled with validation of select target genes/pathways shed new light on the pathogenesis of ovarian GCTs. 

## 2. Materials and Methods

### 2.1. Ethics Statement and Mice

Experimental protocols were approved by the Institutional Animal Care and Use Committee (IACUC) at Texas A&M University. Mice were housed in a climate-controlled vivarium in the Laboratory Animal Resources and Research Facility. Temperature, light cycles, humidity, food, water, bedding, and animal health were closely monitored by experienced veterinary personnel. Animal handling was performed according to the guidelines of the National Institute of Health. All necessary measures were taken to minimize the pain and distress of laboratory animals. Mice harboring constitutively active *TGFBR1* (TGFBR1-CA) were generated as described earlier [9]. 

### 2.2. RNA Isolation and Quantitative Reverse Transcription PCR (qRT-PCR)

Ovaries were collected from both control and TGFBR1-CA mice at the age of 1 month. RNA preparation, reverse transcription (RT) and PCR were performed as described elsewhere [15]. Briefly, ovarian tissues were homogenized in lysis buffer on ice. Total RNA was prepared using QIAGEN RNA isolation kit (Catalog no. 74106). Approximately 500 ng RNA per reaction was used to synthesize the first strand of cDNA. qRT-PCR was performed using iTaq Universal SYBR Green Supermix (Bio-Rad) and oligo primers [16,17] including WNT1 inducible signaling pathway protein 1 (*Wisp1*) (5′-CAGCACCACTAGAGGAAACGA-3′ and 5′-CTGGGCACATATCTTACAGCATT-3′; PrimerBank ID 9256533a1), biglycan (*Bgn*) (5′-TGCCATGTGTCCTTTCGGTT-3′ and 5′-CAGGTCTAGCAGTGTGGTGTC-3′; PrimerBank ID 20137008a1), decorin (*Dcn*) (5′-TCTTGGGCTGGACCATTTGAA-3′ and 5′-CATCGGTAGGGGCACATAGA-3′; PrimerBank ID 6681143a1), cAMP responsive element binding protein 5 (*Creb5*) (5′-AGGATCTTCTGCCGTCTTGAT-3′ and 5′-GCGCAGCCTTCAGTCTCAT-3′; PrimerBank ID 27370070a1), CXXC finger 4 (*Cxxc4*) (5′-CTGCCCGCAGAATCATTCCT-3′ and 5′-CAGACGCCACAGTTGATGAG-3′; PrimerBank ID 365812518c1), and dachshund family transcription factor 1 (*Dach1*) (5′-AGTGGTGGTTCTTGGGATAAGG-3′ and 5′-TGAGAGGATGGCTAACTGGAA-3′; PrimerBank ID 6681129a1). A relative quantification method was conducted using ribosomal protein L19 (*Rpl19*) as an internal control [18]. 

### 2.3. PCR Profiler Array of Oncogenes and Tumor Suppressor Genes

The Mouse Oncogenes & Tumor Suppressor Genes RT² Profiler™ PCR Array (330231 PAMM-502ZA) was purchased from QIAGEN (Germantown, MD, USA). This PCR array contains 84 genes associated with carcinogenesis. The first strand of ovarian cDNA was synthesized for both control and TGFBR1-CA groups (1 mo.; *n* = 3 per group) using RT² First Strand Kit (QIAGEN). Experiments were performed using RT² qPCR Master Mixes and data analyzed using QIAGEN online tools from the GeneGlobe Data Analysis Center (https://geneglobe.qiagen.com/us/analyze, accessed on 9 November 2021). Data normalization was performed using a panel of housekeeping genes identified by the analytic tool. Genes with fold change >2.0 or <−2.0 and adjusted *p*-value < 0.05 were examined.

### 2.4. RNA-Sequencing and Bioinformatics Analysis

RNA-sequencing (RNA-seq) was performed using Illumina NextSeq at the core facility of the Texas A&M Institute for Genome Sciences and Society (TIGSS). Total RNA was isolated from ovaries of control and TGFBR1-CA mice (1 mo.; *n* = 5 per group). RNA quality was assessed by Agilent Bioanalyzer. Data processing and bioinformatics analysis were performed, as detailed previously [19]. Briefly, reads were mapped to the mouse genome using STAR [20]. The number of mapped reads for individual genes was counted using HTSeq-count [21]. EdgeR was applied to determine differential gene expression [22]. Genes with Log_2_ fold change greater than 1 or less than −1 and adjusted *p*-values less than 0.05 were considered to be differentially expressed (DE). Figures were created using the Rgplot and ggplot2 [23]. The original data of RNA-seq were deposited to Gene Expression Omnibus (GEO) (accession number: GSE187015). Enrichr was utilized to identify enriched biological processes, molecular functions, cellular components, and signaling pathways among the DE genes [24,25,26]. For comparative analysis of GCT-associated genes between the mouse and the human, we focused on DE genes related to GCT development, progression, or *FOXL2* (C134W) mutation [11,27,28]. 

### 2.5. Immunohistochemistry

Ovaries were fixed with 10% neutral buffered formalin, embedded in paraffin, and sectioned (5 µm) using a Richard-Allan Microtome. Immunohistochemistry was performed as reported [9]. Briefly, paraffin-embedded sections were mounted on slides, de-waxed in xylene, and rehydrated in ethanol. Heat-induced antigen retrieval was conducted using citrate buffer (pH = 6) and a microwave. Sections were then incubated with 3% H_2_O_2_ to eliminate endogenous peroxidase activity. Sections were blocked using non-immune serum prior to the addition of primary antibodies, which include rabbit anti-non-phospho (active) β-Catenin (Ser33/37/Thr41) IgG (Cell Signaling, Danvers, MA, USA; #8814; 1:1500), and rabbit anti-WISP1 IgG (LSBio, Seattle, WA, USA, LS-C500015, 1:200). Normal rabbit IgG (Cell Signaling; #2729) was included as a negative control. Following incubations with primary antibodies at 4 °C overnight, a secondary biotinylated antibody (BA-1000, Vector laboratories, Burlingame, CA, USA) was added. Immunoreactive signals were amplified using Avidin/Biotin Complex (Vector Laboratories; PK-6100) and developed with NovaRED™ Peroxidase Substrate (Vector Laboratories; SK-4800). 

### 2.6. Statistical Analysis

Quantitative RT-PCR data are expressed as mean ± SEM. Comparison of means from control and TGFBR1-CA mice was performed using a two-tailed *t*-test. A *p*-value < 0.05 was considered statistically significant (* *p*-value < 0.05, ** *p*-value < 0.01, and *** *p*-value < 0.001).

## 3. Results

### 3.1. Transcriptomic Profiles of GCTs Harboring Constitutively Active TGFBR1 

**Figure 1:**
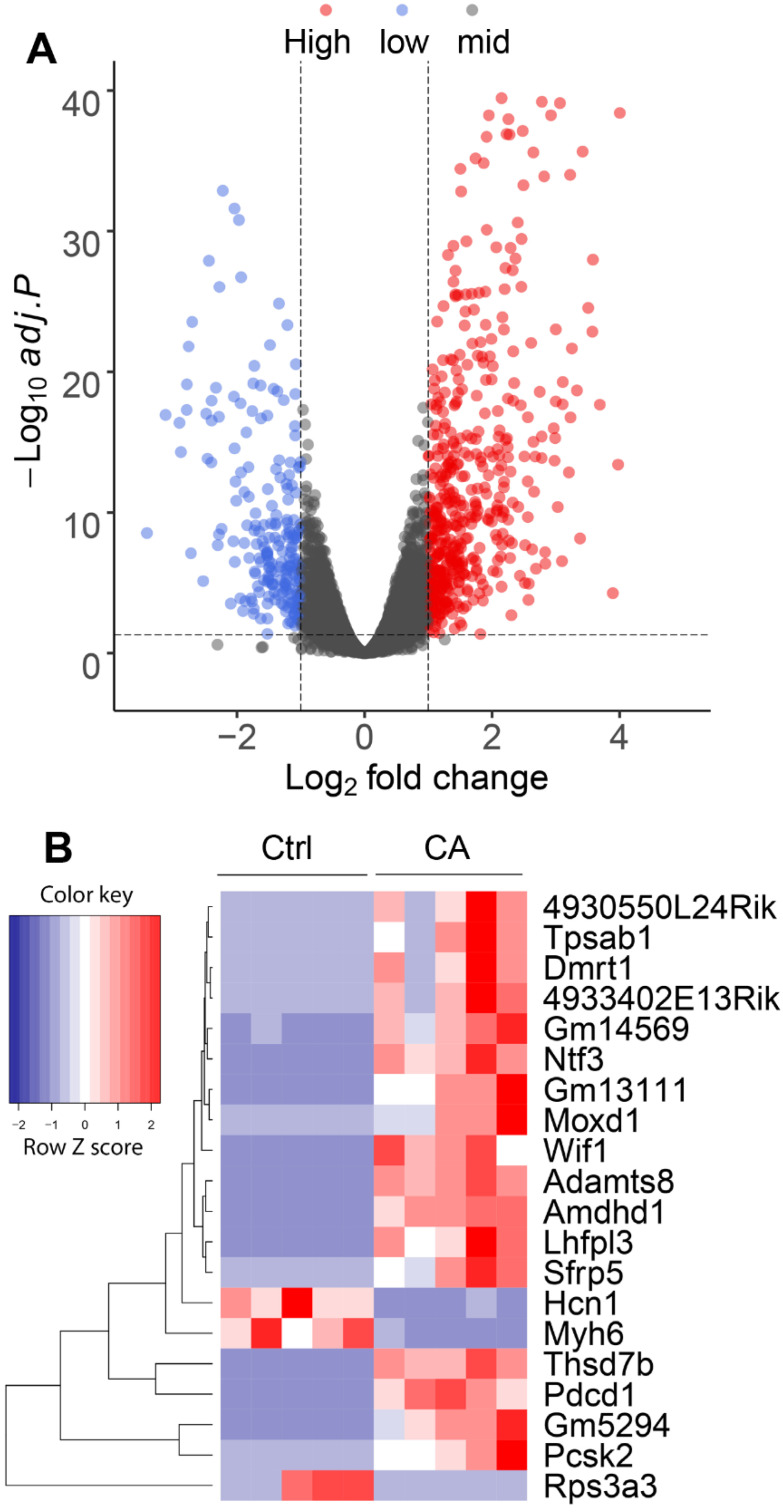
RNA-seq analysis of ovaries from TGFBR1-CA and control mice at the age of 1 month. (**A**) Volcano plot depicting genes identified by RNA-seq. Genes with Log_2_ fold change greater than 1 (red) or less than −1 (blue) and adjusted *p*-values less than 0.05 were defined as DE genes. A total of 715 DE genes were identified, with 499 upregulated genes and 216 downregulated genes in the ovaries of TGFBR1-CA mice compared with age-matched controls. *n* = 5 per group. (**B**) Heatmap of the top 20 DE genes. Heatmap was generated using row Z scores. A full list of DE genes is shown in Supplementary Table S1.

To begin understanding the molecular changes in GCT development induced by overactivation of TGFBR1, we first performed a PCR profiler array analysis targeting 84 oncogenes and tumor suppressors using ovaries from TGFBR1-CA mice and controls at the age of 1 month when ovarian GCTs began to develop. Three DE genes (fold change > 2.0 or <−2.0 and adjusted *p*-value < 0.05) were identified in TGFBR1-CA ovaries (Figure S1A,B). S100A4 belongs to S100 family proteins containing calcium-binding motifs. Upregulation of *S100a4* in TGFBR1-CA ovaries is likely associated with tumor formation, as *S100a4* is implicated in multiple cellular and inflammatory events that promote tumorigenesis [29]. *Cdkn2a* encodes a cell-cycle inhibitor and is a well-known tumor suppressor [30]. It has been demonstrated that loss of CDKN2A and/or CDKN2B expression occurs in ~58% adult GCTs [31]. However, *Cdkn2a* was upregulated in TGFBR1-CA ovaries compared with controls, possibly serving as a feedback mechanism that counteracts tumorigenesis. Interpretation of the results should be cautious as mutation/overexpression of tumor suppressors/oncogenes do not appear to be directly associated with ovarian GCT development [2]. In addition, the assay is limited to a panel of oncogenes and tumor suppressors, with little information garnered on gene ontology and tumor-associated signaling cascades. Therefore, we undertook RNA-seq, an unbiased approach to identify the global transcriptomic changes during ovarian GCT development using ovaries from TGFBR1-CA and control mice at the age of 1 month. Independent sample sets were used for the RNA-seq experiment. Bioinformatics analysis identified 715 DE genes (Supplementary Table S1), with the volcano plot and heatmap of the top 20 significantly regulated genes depicted (Figure 1A,B). The functions of most of these genes remain unclear in ovarian GCTs. However, programmed cell death 1 (*Pdcd1*) [32], proprotein convertase subtilisin/kexin type 2 (*Pcsk2*) [33], and LHFPL tetraspan subfamily member 3 (*Lhfpl3*) [34] are known to be associated with cancer development. Interestingly, the tumor suppressor ADAM metallopeptidase with thrombospondin type 1 motif 8 (*Adamts8*) was also upregulated. Moreover, the expression of two WNT signaling inhibitors, the secreted frizzled related protein 5 (*Sfrp5*) and WNT inhibitory factor 1 (*Wif1*), was increased in TGFBR1-CA ovaries versus the controls. Increased expression of tumor suppressors and WNT inhibitors likely serves as a negative feedback that attenuates tumorigenesis and the activation of WNT signaling important for ovarian GCT development. 

**Figure 2:**
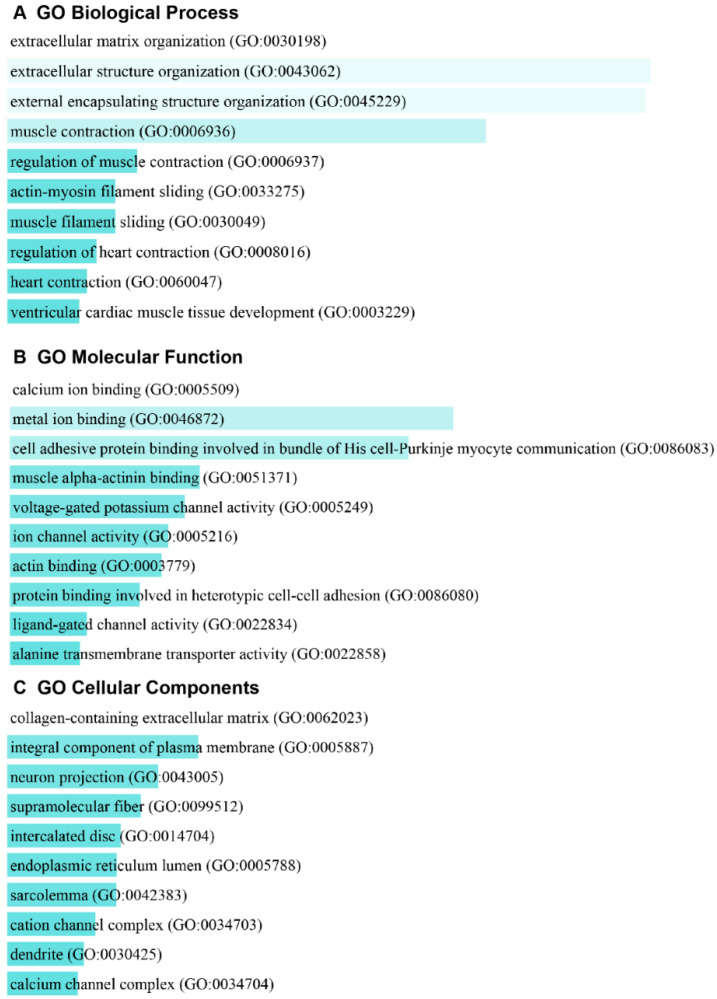
GO analysis of differentially expressed genes in ovaries between TGFBR1-CA and control mice. (**A**–**C**) Top 10 GO biological processes, molecular functions, and cellular components revealed by Enrichr analysis using DE genes from RNA-seq. Full lists are shown in Supplementary Tables S2–S4.

To identify enriched biological annotations among the DE genes, we performed Enrichr gene ontology analysis of the biological processes, molecular functions, and cellular components. The top 10 GO biological processes (Figure 2A), molecular functions (Figure 2B), and cellular components (Figure 2C) are shown as bar graphs and the complete information was included as Supplementary Tables S2–S4. Extracellular matrix organization (GO: 0030198) and extracellular structure organization (GO: 0043062) were the top two enriched biological processes (Figure 2A). This finding supports the critical involvement of the extracellular matrix (ECM) in controlling tumor growth and malignancy [35]. Some top molecular functions were associated with calcium ion binding (GO: 0005509), metal ion binding (GO: 0046872), voltage-gated potassium channel activity (GO: 0005249), ion channel activity (GO: 0005216), and actin binding (GO: 0003779) (Figure 2B). The analysis also revealed molecular alterations consistent with key roles of calcium ions [36] and actin cytoskeleton [37] in tumor development (Figure 2B). As interactions between ion channels and cytoskeleton influence tumor development [38], current findings highlight a plausible role of ion channels and actin cytoskeleton in ovarian GCT development. Collagen-containing extracellular matrix (GO: 0062023) was the most significantly associated cellular component (Figure 2C), lending support to the importance of collagen in tumor microenvironment and tumor development [39]. Taken together, the gene ontology analysis provides biological, molecular, and cellular characteristics of GCT development in TGFBR1-CA mice. 

### 3.2. Identification of Signaling Pathways and Regulators Associated with GCT Development

**Figure 3:**
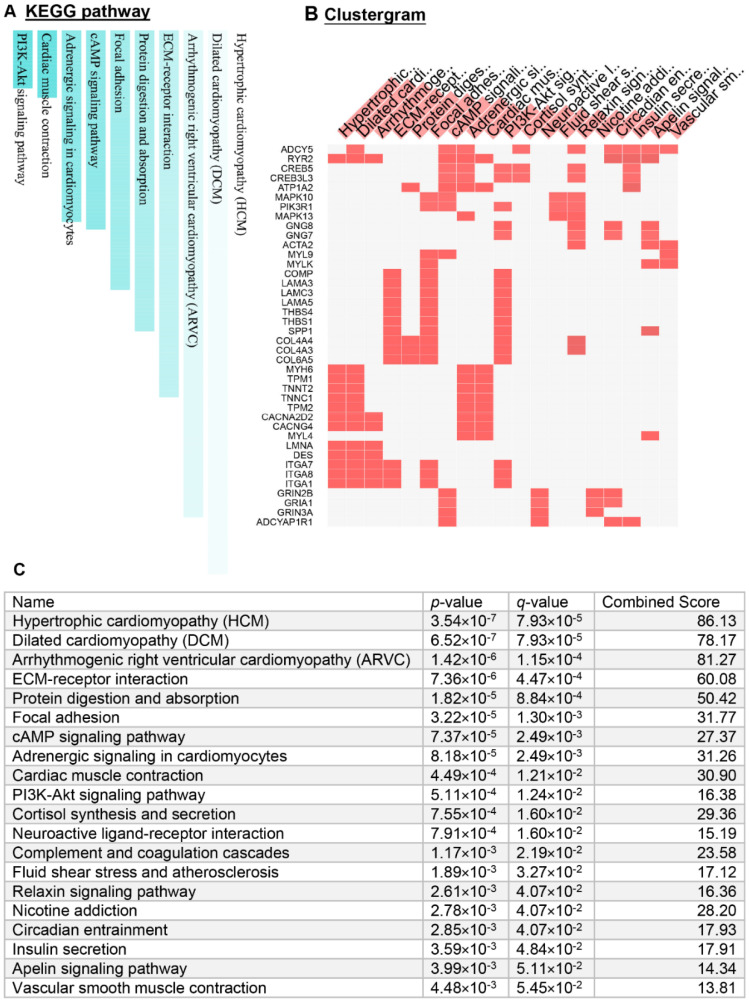
KEGG pathways enriched among DE genes in ovaries between TGFBR1-CA and control mice at the age of 1 month. (**A**) The top 10 differentially regulated signaling pathways. (**B**) Clustergram depicting DE genes associated with the top 20 differentially regulated signaling pathways. Note that only 40 out of 715 DE genes are shown. (**C**) Statistical parameters of the top 20 significantly regulated pathways. A full list of pathways is shown in Supplementary Table S5.

To explore potential gene networks regulated by constitutively activated TGFβ signaling, we first performed a KEGG pathway analysis using the Enrichr KEGG mouse 2019 database (Figure 3 and Supplementary Table S5). The bar graph and clustergram of the top-ranked pathways are shown in Figure 3A,B, respectively. Detailed statistical parameters for these pathways are depicted in Figure 3C. Among the top 20 signaling pathways, the cyclic AMP signaling pathway and PI3K-Akt pathway have known functions within GCTs [40,41]. However, the functions of the other pathways in ovarian GCT development were largely unknown. Of note, eight pathways were associated with cardiovascular functions/diseases including hypertrophic cardiomyopathy (HCM), dilated cardiomyopathy (DCM), arrhythmogenic right ventricular cardiomyopathy (ARVC), adrenergic signaling in cardiomyocytes, cardiac muscle contraction, fluid shear stress and atherosclerosis, apelin signaling pathway, and vascular smooth muscle contraction. The pathways were identified based on the differential expression of a cohort of genes associated with angiogenesis, cell structure, and cell motility (Figure 3B,C and Supplementary Table S5). Pathways linked to metabolism include protein digestion and absorption and cortisol synthesis and secretion. Two signaling pathways, ECM-receptor interaction and focal adhesion, are associated with crosstalk between extracellular matrix and cancer cells. Dysregulation of circadian entrainment associated signaling pathway was also found (Figure 3B,C and Supplementary Table S5).

Several pathways that have been implicated in the development of other cancer types were enriched in TGFBR1-CA ovaries (Figure 3C and Supplementary Table S5): (1). Neuroactive ligand-receptor interaction. Interactions between tumor cells and neuronal tissues link to cancer development [42]. Little is known about this pathway in ovarian GCTs. Of particular interest was the upregulation of neuromedin U receptor 2 (*Nmur2*), which encodes a receptor for neuromedin U (NMU), originally recognized as an appetite-modulating peptide. NMU has now been recognized as an emerging player in tumorigenesis [43]. (2). Complement and coagulation cascades. The complement system mediates innate immunity and its activation within the tumor microenvironment promotes tumor development [44]. (3). Relaxin signaling. Relaxin is known as a diagnostic biomarker for epithelial ovarian cancer [45]. However, the knowledge bridging relaxin signaling, TGFβ signaling, and ovarian GCTs is very limited. (4). Insulin signaling. Insulin signaling influences carcinogenesis [46], with little information available in ovarian GCTs. Thus, our transcriptomic profiling combined with bioinformatics analysis revealed pathways potentially associated with the development of GCTs induced by TGFβ signaling activation.

**Figure 4:**
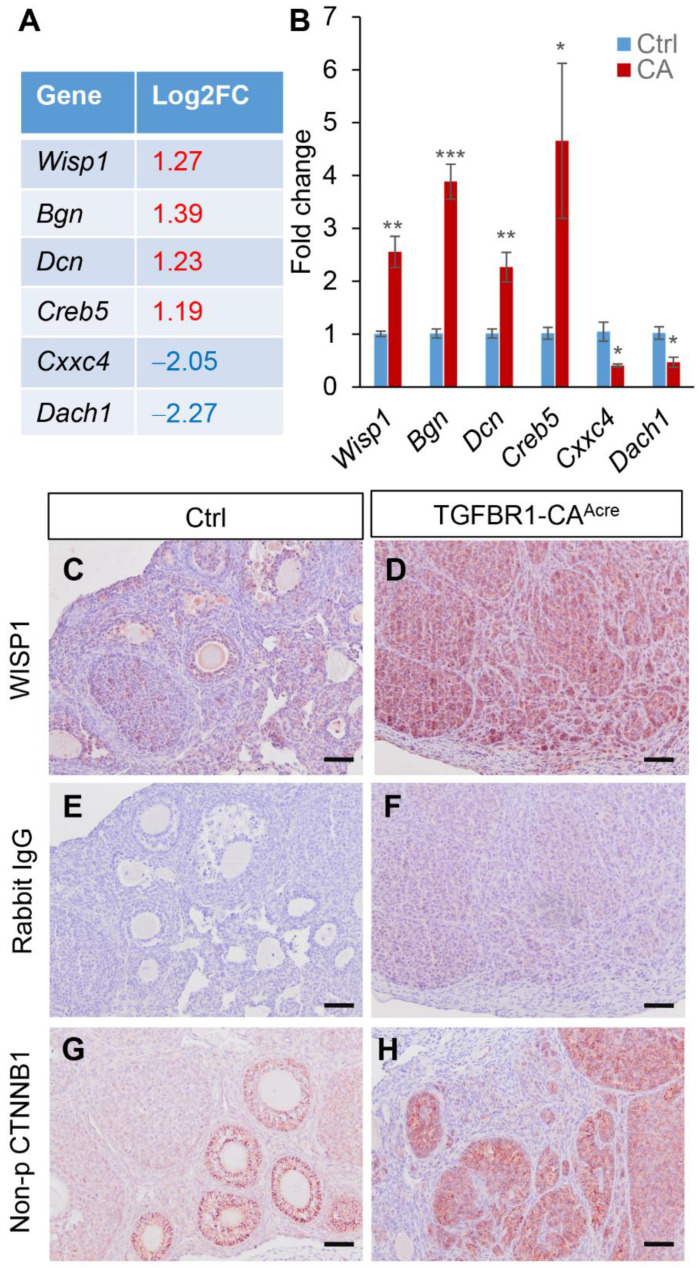
Upregulation of *Wisp1* and WNT signaling activation in TGFBR1-CA ovaries. (**A**) Log_2_ fold changes of a subset of WNT-related genes from RNA-seq analysis. (**B**) qRT-PCR analysis of *Wisp1*, *Bgn*, *Dcn*, *Creb5*, *Cxxc4*, and *Dach1* in ovaries from TGFBR1-CA and control mice at the age of 1 month. *n* = 4. * *p*-value < 0.05, ** *p*-value < 0.01, and *** *p*-value < 0.001. (**C**–**H**) Immunohistochemical staining of WISP1 and non-phospho CTNNB1 in ovaries from TGFBR1-CA and control mice at the age of 1 month. Isotype-matched rabbit IgG was included as a negative control. *n* = 3. Scale bar = 50 µm (**C**–**H**).

WNT pathway is known to interact with TGFβ signaling and plays a key role in GCT development [3,9,47]. Unexpectedly, the Enrichr KEGG pathway analysis failed to identify the enrichment of WNT signaling in TGFBR1-CA ovaries. To determine whether WNT signaling components were dysregulated, we examined our DE gene list and found the upregulation of the WNT signaling-associated oncogene *Wisp1* (Log_2_FC = 1.27) and several WNT signaling-associated genes in TGFBR1-CA ovaries, supporting WNT signaling activation. These genes include *Bgn* (Log_2_FC = 1.39), *Dcn* (Log_2_FC = 1.23), *Creb5* (Log_2_FC = 1.19), *Cxxc4* (Log_2_FC = −2.05), and *Dach1* (Log_2_FC = −2.27) (Figure 4A and Supplementary Table S1). WISP1 belongs to the CCN family (also known as CCN4) and is inducible by WNT signaling [48]. BGN and DCN are binding factors of WISP1 [49], and CREB5 has been associated with TGFβ and WNT signaling [50]. CXXC4 and DACH1 are WNT antagonists [51,52]. Using qRT-PCR, we verified the transcript changes of these genes in TGFBR1-CA ovaries versus controls (Figure 4B). Consistent with the mRNA expression of *Wisp1*, WISP1 protein was strongly localized to TGFBR1-CA ovaries, particularly in the tumor foci (Figure 4D), in contrast to the limited immunoreactive signals in control ovaries (Figure 4C). Negative controls of immunostaining are shown in Figure 4E,F. As mentioned earlier, a few WNT antagonists, including *Sfrp5* and *Wif1*, were also upregulated in TGFBR1-CA ovaries. Thus, it is necessary to further determine the status of WNT signaling in TGFBR1-CA ovaries. To this end, we conducted immunostaining of non-phospho β-Catenin (CTNNB1; the effector of WNT signaling). Non-phospho CTNNB1 (i.e., active CTNNB1) was detected in tumor tissues of TGFBR1-CA ovaries (Figure 4H), whereas its expression was restricted to early stage follicles in the control ovaries (Figure 4G). Collectively, our studies indicated active WNT signaling in early GCTs and identified WISP1 as a potential novel signaling molecule in GCT development.

### 3.3. Identification of Potential Clinically Relevant Regulators of Ovarian GCT Development

**Figure 5:**
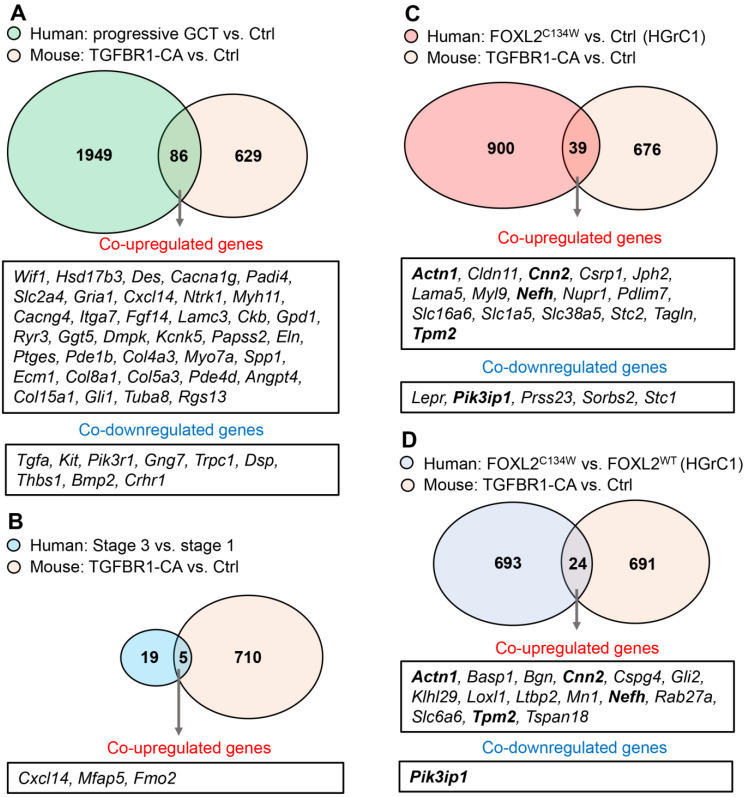
Identification of common regulators of ovarian GCT development between mice and humans. (**A**) Comparison of DE genes in TGFBR1-CA ovaries with those between the human progressive GCT and normal ovaries. Gene expression of human GCTs was previously reported [28]. (**B**) Comparison of DE genes in TGFBR1-CA ovaries with those between advanced stage and early stage GCTs. Identification of tumor stage-associated genes was previously documented [27]. (**C**,**D**) Comparison of differentially expressed genes in TGFBR1-CA ovaries with those between FOXL2^C134W^ and vehicle or FOXL2^WT^-transfected HGrC1. An immortalized human granulosa cell line was used to reveal genes associated with FOXL2 mutation [11]. Overlapping genes between panels (**C**,**D**) are bolded.

To determine the potential translational value of our mouse model, we compared our DE genes with those associated with human granulosa cell tumor development or FOXL2 mutation. First, comparison of DE genes with those between the human progressive GCT and normal ovaries [28] identified 36 co-upregulated genes and 9 co-downregulated genes (Figure 5A). Among the co-upregulated genes, the functions of most, if not all, genes in ovarian GCTs are unknown. Chemokine (C-X-C motif) ligand 14 (CXCL14) is a chemokine with a wide variety of activities including the regulation of immune cell migration and antimicrobial immunity [53]. Secreted phosphoprotein 1 (*Spp1*) is expressed in ovarian granulosa cells [54] and promotes ovarian cancer progression [55]; however, its role in ovarian GCT development is unclear. Upregulation of several collagen genes (i.e., *Col4a3*, *Col8a1*, *Col5a3*, and *Col15a1*) reinforced the role of ECM in GCT pathogenesis. Reduction of *Bmp2* expression is consistent with the tumor suppressive role of BMP signaling in the ovarian GCT formation [4].

Second, we compare our DE gene list with the 24 genes differentially expressed between the advanced stage GCT and early stage GCTs reported previously [27]. The analysis identified three co-upregulated genes that include *Cxcl14*, the flavin containing dimethylaniline monoxygenase 2 (*Fmo2*), and the microfibrillar-associated protein 5 transcript variant 1 (*Mfap5*) (Figure 5B). MFAP5 is a component of microfibrils of the ECM [56]. Thus, upregulation of *Mfap5* may promote ovarian GCT progression via interacting with other ECM signaling pathways. FMO2 is a NADPH-dependent enzyme implicated in oxidation reactions and plays a possible role in lung adenocarcinoma [57].

Third, our DE gene list was compared with genes impacted by transfection of the FOXL2^C134W^ construct in a human granulosa cell line, HGrC1 [11]. We identified 16 co-upregulated genes and 5 co-downregulated genes (Figure 5C). The co-upregulated genes included, but were not limited to, cytoskeleton-associated genes [actinin alpha 1 (*Actn1*), calponin 2 (*Cnn2*), PDZ and LIM domain 7 (*Pdlim7*) and tropomyosin 2 (*Tpm2*)], junction-related genes [claudin 11 (*Cldn11*) and junctophilin 2 (*Jph2*)], and a cancer-associated gene, the nuclear protein 1 transcriptional regulator (*Nupr1)* [58]. In addition, we compared our DE genes with those between FOXL2^C134W^-transfected and FOXL2^WT^-transfected HGrC1 cells. In this case, 15 co-upregulated genes and 1 co-downregulated gene were found (Figure 5D). A few common genes (bolded) were observed between the two comparisons [i.e., *Actn1*, *Cnn2*, *Nefh*, *Tpm2*, and phosphoinostitide-3-kinase interacting protein 1 (*Pik3ip1*)]. As PIK3IP1 negatively regulates PI3K signaling [59], downregulation of *Pik3ip1* is consistent with the derepression of PI3K signaling in GCT development [60]. Thus, the identification of commonly regulated genes between human GCTs or FOXL2^C134W^-expressing granulosa cells and TGFBR1-CA ovaries suggests a potential translational value of our mouse model in GCT research.

Finally, the aforementioned co-upregulated and co-downregulated genes between our TGFBR1-CA mice and human GCTs are likely meaningful in determining tumorigenic mechanisms of ovarian GCTs between species. However, genes that were expressed in different patterns between human and mouse GCTs (Supplementary Table S6) may be useful in dissecting the distinct mechanisms of GCT development in the mouse and the human. For instance, ecto-5′-nucleotidase (*Nt5e*) was upregulated in the ovary of TGFBR1-CA mice; however, it was downregulated in human progressive GCT tissues. Thus, *Nt5e* may play different roles in human and mouse GCTs. Differences in molecular signatures between human and mouse GCTs should be considered when extrapolating findings from the mouse model.

## 4. Discussion

Understanding of the molecular pathogenesis of ovarian GCTs is far from complete. In the present study, we took advantage of our previously established TGFBR1-CA mouse model [9] to explore the molecular basis of ovarian GCT development in the context of TGFβ signaling activation. GCTs were examined at the age of 2 months in our previous study to determine histological features, as gross ovarian GCTs develop by 2 months of age in TGFBR1-CA mice [9]. In contrast, we used ovaries from 1-month-old mice in this study to identify the molecular mechanisms during early tumorigenesis. Whole ovaries have been used to determine mechanisms of ovarian GCT development in mice containing a dominant-stable CTNNB1 and KRAS activation/PTEN depletion [41].

Our RNA-seq analysis yielded a wealth of information. By performing gene ontology analysis using Enrichr [24,25,26], we identified biological processes involved in ECM-associated activities. ECM composition and topography are known to regulate cancer progression [61]. The roles of multiple ECM components have been reviewed in the epithelial ovarian cancer [62]. Collagen is a tumor microenvironment component that impacts cancer cell behavior via interaction with multiple signaling molecules [39]. Notably, several collagen-associated genes such as *Col15a1*, *Col5a3*, *Col4a3*, *Col8a1* were upregulated in TGFBR1-CA ovaries. *Col4a3* expression is associated with the poor prognosis of lung cancer patients after receiving chemotherapy [63], and *Col8a1* [64], *Col5a3* [65], and *Col15a1* [66] are involved in tumor growth and development. In addition to collagen, elastin (*Eln*) and laminin 5 (*Lama5*) were also upregulated in TGFBR1-CA ovaries. Both genes have been linked to cancer development [67,68]. Of note, lysyl oxidase (LOX) family members participate in crosslinking and stabilizing collagen and elastin, regulate cell growth, and are elevated in invasive tumors [69]. Thus, the upregulation of ECM-associated genes highlights their potential roles in tissue remodeling and tumor cell-ECM crosstalk during ovarian GCT development. The ECM-related genes differentially expressed between TGFBR1-CA mice and controls may be further exploited to determine their potential value in tumor progression and diagnosis.

Of note, only three DE genes with relatively low expression levels were identified by PCR profiler array but not RNA-seq. The use of independent sample sets for PCR profiler array and RNA-seq is expected to increase the rigor of the experiments, but could also bring more variations due to individual sample differences. In addition, the RNA-seq and PCR profiler array use different methods to determine gene expression. While RNA-seq counts the reads of RNA transcripts, PCR profiler array relays on measuring fluorescence intensity. It was also noted that among the 89 genes, including the 84 oncogenes/tumor suppressors and 5 housekeeping genes tested by the predesigned PCR profiler array, ~87% of them were detected by RNA-seq analysis (Supplementary Table S7), indicating that our RNA-seq data processing provides reliable gene expression profiling. Moreover, the results of the PCR profiler array indicate that an unbiased RNA-seq analysis is necessary to complement the PCR profiler array, which has a very limited assay scope.

Activation of WNT/CTNNB1 pathway is involved in the development of GCTs; mice expressing a dominant stable CTNNB1 mutant in ovarian granulosa cells develop GCTs [3]. Consistently, the amplification of R-spondin1 that activates WNT signaling leads to GCT formation [70,71]. Guided by the RNA-seq finding, we revealed ovarian WNT signaling activation in TGFBR1-CA mice by demonstrating the localization of non-phosphorylated active CTNNB1 to the tumor foci. A new finding is the identification of *Wisp1*, a direct target of WNT signaling [72], and its associated factors as potential regulators of ovarian GCT formation. While the reduced expression of WNT antagonists (e.g., *Dach1* and *Cxxc4*) may facilitate WNT signaling activation, it is counterintuitive that *Sfrp5* and *Wif1* were upregulated in TGFBR1-CA ovaries. Both genes are known to antagonize WNT signaling [73,74]. It is unclear whether increased expression of WNT antagonists acts as a negative feedback to restrict WNT signaling activation or merely reflects a consequence of TGFBR1 activation in vivo.

Our bioinformatics analysis provided new insights into signaling pathways involved in GCT development in the TGFBR1-CA mouse model. Many of the top 20 dysregulated pathways are associated with cardiovascular diseases due to the changes of genes related to angiogenesis, cell structure, and cell motility that also occur during tumor development. Critical genes associated with angiogenesis and vascular functions include angiotensin-I-converting enzyme (*Ace*), vascular cell adhesion molecule 1 (*Vcam1*), platelet derived growth factor subunit A (*Pdgfa*), and apelin receptor (*Aplnr*). ACE is expressed in rat granulosa cells and is an important regulator of tumor development [75,76]. Increased serum ACE levels have been detected in epithelial ovarian cancer patients [77]. Both VCAM1 and PDGFA are associated with angiogenesis and involved in cancer development [78,79]. Abnormal expression of the above genes in TGFBR1-CA ovaries indicates dysregulated vascular functions during ovarian GCT development. Apelin via its receptor APLNR drives pro-angiogenic effects during tumorigenesis [80]. However, *Aplnr* was downregulated in TGFBR1-CA ovaries. Cell structure and motility related genes include tropomyosin 1 (*Tpm1*), *Tpm2*, and troponin C1, slow skeletal, and cardiac type (*Tnnc1*). *Tpm1/2* and *Tnnc1* were upregulated in TGFBR1-CA ovaries. TPM regulates actin filaments and is associated with cancer cell transformation [81]. TNNC is associated with ovarian cancer cell motility/invasion [82]. Thus, upregulation of *Tpm* and *Tnnc* may reflect changes in cell structure/motility during GCT development. Cancer development is known to alter the metabolism [83]. Metabolism-associated pathways were identified among the top 20 dysregulated pathways. Solute carrier family 1 member 5 (*Slc1a5*) and *Slc7a8* encode transmembrane transporters. SLC1A5 plays a role in lung cancer [84] and SLC7A8 is involved in breast cancer development [85]. The upregulation of these transporter-associated genes in TGFBR1-CA ovaries suggests altered metabolic status during GCT development.

Besides the aforementioned key tumorigenic processes, several additional pathways merit further discussion. Relaxin is a hormone belonging to the insulin superfamily that acts through its receptor relaxin family peptide receptor 1 (RXFP1/LGR7) [86]. Relaxin signaling promotes cancer progression and invasion [87,88]. However, the role of relaxin in ovarian GCTs remains elusive. A well characterized role of relaxin signaling is the antifibrotic effect on connective tissue remodeling [89]. In TGFBR1-CA ovaries, multiple fibrosis-associated genes [actin alpha 2, smooth muscle, aorta (*Acta2*), mitogen-activated protein kinase 10 (*Mapk10*), *Creb3l3*, *Col4a3*, matrix metallopeptidase 9 (*Mmp9*), and *Creb5*] were upregulated, consistent with TGFBR1 activation. Tumor-associated fibroblasts regulate tumor growth and progression. Indeed, nano-targeted relaxin attenuates pancreatic tumor growth and fibrosis [90]. Insulin signaling maintains glucose homeostasis [91] and is involved in cancer development and progression [92]. Adenylate cyclase 5 (*Adcy5*) encodes an enzyme important for normal insulin release [93]. The expression of *Adcy5* was increased in TGFBR1-CA ovaries, suggestive of altered insulin signaling. Our pathway analysis also identified the enrichment of a constituent of innate immunity, the complement system that is associated with tumor microenvironment and tumor development [44]. Recent studies demonstrate the complement as a double-edged sword, owing to its anti-tumoral and pro-tumoral functions [94]. Complement C1q (C1q) activates the classical pathway via ligand binding to regulate immune responses [95]. Our RNA-seq analysis revealed the upregulation of *C1qa*, *C1qb* and *C1qc*, and C7 in TGFBR1-CA ovaries. As little is known about the tumor microenvironment in ovarian GCTs, defining the role of complements in ovarian GCTs represents a new arena that requires further research efforts. Collectively, our pathway analysis not only revealed key tumorigenic processes (e.g., angiogenesis and metabolism) in ovarian GCT development, but also identified pathways and genes whose functions have not been studied in ovarian GCTs. Further efforts defining the functional requirement of these candidate genes/pathways in GCT development may benefit the development of new therapeutic modalities.

Additional data mining efforts have pinpointed a cohort of genes that were co-regulated in both mouse and human GCTs. Many co-regulated genes are important for tumor development, albeit their specific roles in ovarian GCTs remain to be established. As examples, chondroitin sulfate proteoglycan 4 (*Cspg4*) regulates tumor growth and metastasis [96]. Gamma-glutamyltransferase 5 (*Ggt5*) promotes tumor growth in the lung [97]. Extracellular matrix protein 1 (*Ecm1*) is highly expressed in multiple malignant epithelial tumors [98]. Additionally, desmoplakin (*Dsp*) is a tumor suppressor whose deletion provokes tumor invasion in a pancreatic tumor model [99]. Among the co-upregulated genes, several of them are known targets of TGFβ signaling. Nuclear protein 1, the transcription regulator (*Nupr1*), is inducible by TGFβ signaling, as it contains a TGFβ-responsive element in the promoter region [100]. NUPR1 has been involved in the development of multiple types of cancers [58]. Transgelin (*Tagln*) is also a TGFβ signaling regulated gene [101] and its upregulation predicts a poor overall survival outcome in colorectal cancer patients [102]. Moreover, TGFβ signaling regulates the expression of 3′-phosphoadenosine 5′-phosphosulfate synthase 2 (*Papss2*) [103], a gene related to breast cancer cell migration [104]. These findings reinforce the importance of TGFβ signaling activation in GCT development. Another interesting finding is the increased expression of several cytoskeletal proteins/regulators in both the mouse and the human GCTs. The actin cytoskeleton is crucial for cellular homeostasis [37], and regulation of cytoskeletal protein expression is implicated in granulosa cell differentiation [105]. Thus, dysregulation of cytoskeletal genes may be associated with GCT development via affecting granulosa cell differentiation.

Our results also demonstrated increased expression of *Cxcl14* and *Mfap5.* CXCL14 is a chemokine that regulates immune and inflammatory functions [53], but its role in ovarian GCTs is unclear. Consistent with a role in tumor microenvironment, MFAP5 is present in cancer-associated fibroblasts of high-grade serous ovarian cancer and interacts with tumorigenic signaling [82]. Future characterization of the spatiotemporal dynamics and potential roles of these genes in the ovary, particularly in granulosa cells, will be instrumental in defining their tumor-related functions. Of note, limitations exist in the comparative approach performed to identify commonly regulated genes between mouse and human GCTs. The differentially expressed genes from the transcriptomic analyses of the two human GCT studies were derived from the comparison of progressive GCTs to normal ovaries or advanced stage GCTs to early stage GCTs [27,28]. Therefore, these genes are likely associated with tumor progression or malignancy. In contrast, differentially expressed genes from our datasets may be more relevant to tumor initiation and/or formation.

The small number of overlapping genes revealed by the comparative analysis may be caused by the following reasons: (1). Differences in tumor stages (i.e., tumor formation/ initiation vs. tumor progression). Of note, the human study comparing advanced stage GCTs with early stage GCTs has only identified 24 DE genes [27]. Therefore, it is not surprising that only a limited number of genes were identified between the mouse and human GCTs in this case. (2). Sampling methods (i.e., tumor tissues or cell lines vs. whole ovaries): Tumor tissues and GCT cell lines have been used for the transcriptomic analyses of human GCTs, while ovaries were used for the RNA-seq analysis of TGFBR1-CA mice. In addition, the different platforms used to profile the transcriptome (microarray vs. RNA-seq) and the intrinsic difference in gene signatures of the models may also contribute to the small number of overlapping DE genes identified in the comparative analysis. It is reasonable that mouse models targeting different genes/pathways have distinct gene signatures. Supporting this notion, only a limited number of genes were identified when DE genes in TGFBR1-CA ovaries were compared with those in *Smad1/5/8* conditional mutant mice [4] (Supplementary Figure S2). Although studies using existing mouse models of GCTs have identified important signaling events critical for the pathogenesis of GCTs, it is worthwhile mentioning that all current mouse models do not mimic *FOXL2* mutation (C134W), the genetic hallmark of human ovarian GCTs. Thus, it will be important to develop a mouse model that recapitulates the genetic mutation of human GCTs. Nevertheless, commonly regulated genes between the mouse and human GCTs may be valuable and their functions can be further interrogated. On the other hand, exploiting the non-overlapping genes may help identify the distinct mechanisms governing ovarian GCT development in the mouse and the human.

## 5. Conclusions

This study has revealed the molecular signature of ovarian GCTs in a mouse model that harbors a constitutive activation of TGFBR1. Comparative analyses of our data with genes dysregulated in human GCTs or granulosa cells overexpressing a mutant FOXL2 have identified some common genes altered in both conditions. The findings can be further exploited to understand the functions of these genes and their potential implications in ovarian GCT development.

## Data Availability

Data are included in the article and Supplementary Materials.

## Supplementary Materials

The following are available online at https://www.mdpi.com/article/10.3390/cancers14092184/s1, Table S1: Differentially expressed genes in ovaries between TGFBR1-CA and control mice. Table S2: GO biological processes identified by Enrichr. Table S3: GO molecular functions identified by Enrichr. Table S4: GO cellular components identified by Enrichr. Table S5: KEGG pathways enriched in TGFBR1-CA ovaries versus controls. Table S6: Overlapping DE genes in opposite directions between TGFBR1-CA ovaries and human ovarian GCTs or granulosa cells overexpressing a mutant FOXL2. Table S7: Detectability of genes on PCR profiler array by RNA-seq. Figure S1: PCR profiler array analysis using ovaries from TGFBR1-CA and control mice. Figure S2: Overlapping DE genes between TGFBR1-CA ovaries and *Smad1/5/8* mutant GCTs.
