# Supplementary material for: Transcriptomic Profiling of Gene Expression Associated with Granulosa Cell Tumor Development in a Mouse Model
Source: Cancers (Basel). 2022 Apr 27;14(9):2184. doi: 10.3390/cancers14092184 (PMC9105549; doi:10.3390/cancers14092184)
Supplement: Supplementary file 1 [file cancers-14-02184-s001.zip › cancers-1620022-supplementary/Supplementary/Figure S1.pdf]

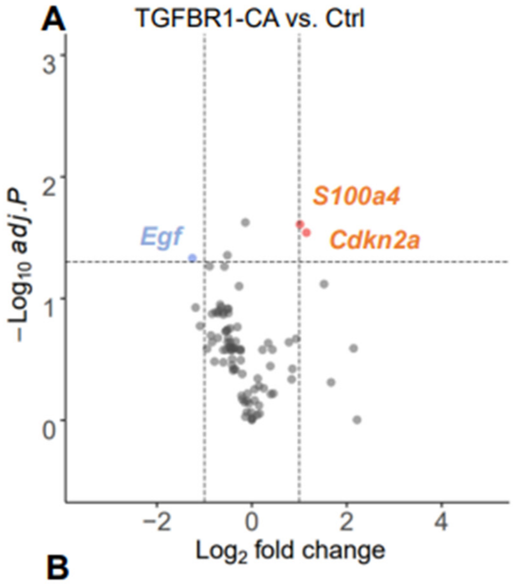

B

Gene	Fold	Adjusted *p*-value
Cdkn2a	2.22	0.0287
S100a4	2.02	0.0246
Egf	-2.38	0.0466

Figure S1: PCR profiler array analysis using ovaries from TGFBR1-CA and control mice. (**A**) Volcano plot showing the result of PCR profiler array using ovaries from TGFBR1-CA mice and age-matched controls at the age of 1 month. Three genes with fold changes > 2 or < -2 and adjusted *p*-values < 0.05 were identified. n = 3 per group. The figure was generated using EnhancedVolcano package in R. (**B**) Fold changes and adjusted *p*-values of DE genes identified by PCR profiler array.
