# Supplementary material for: Transcriptomic Profiling of Gene Expression Associated with Granulosa Cell Tumor Development in a Mouse Model
Source: Cancers (Basel). 2022 Apr 27;14(9):2184. doi: 10.3390/cancers14092184 (PMC9105549; doi:10.3390/cancers14092184)
Supplement: Supplementary file 1 [file cancers-14-02184-s001.zip › cancers-1620022-supplementary/Supplementary/Figure S2.pdf]

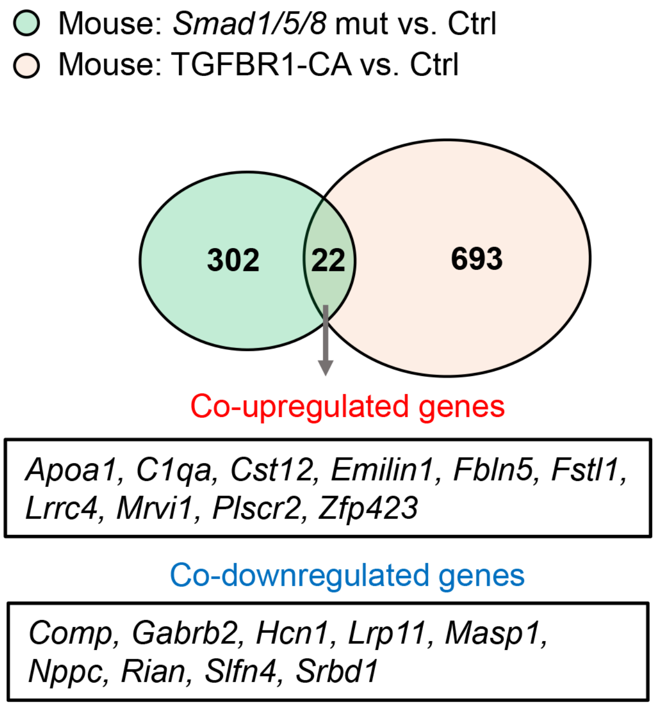

Figure S2. Overlapping DE genes between TGFBR1-CA ovaries and *Smad1/5/8* mutant GCTs.
